# Implementation and User Satisfaction of a Comprehensive Telemedicine Approach for SARS-CoV-2 Self-Sampling: Monocentric, Prospective, Interventional, Open-Label, Controlled, Two-Arm Feasibility Study

**DOI:** 10.2196/57608

**Published:** 2024-12-11

**Authors:** Florian Voit, Johanna Erber, Silvia Egert-Schwender, Michael Hanselmann, Michael Laxy, Victoria Kehl, Dieter Hoffmann, Samuel D Jeske, Thomas Michler, Ulrike Protzer, Florian Kohlmayer, Roland M Schmid, Christoph D Spinner, Simon Weidlich

**Affiliations:** 1 Clinical Department for Internal Medicine II - Department of Clinical Medicine, TUM School of Medicine and Health, Technical University of Munich, Munich, Germany; 2 Muenchner Studienzentrum, TUM School of Medicine and Health, Technical University of Munich, Munich, Germany; 3 Professorship of Public Health and Prevention, Department Health and Sport Sciences, TUM School of Medicine and Health, Technical University of Munich, Munich, Germany; 4 Institute of Virology, TUM School of Medicine and Health, Technical University of Munich, Munich, Germany; 5 Professorship of Medical Informatics, Institute for Artificial Intelligence and Informatics in Medicine, TUM School of Medicine and Health, Technical University of Munich, Munich, Germany

**Keywords:** telemedicine, self-sampling, SARS-CoV-2, user satisfaction, user, implementation, acute respiratory syndrome, respiratory syndrome, coronavirus, self-sampling, monocentric, prospective, interventional, open-label, two-arm feasibility study, innovative, application, healthcare, treatment, mobile phone, pandemic control, health care

## Abstract

**Background:**

The universal availability of smartphones has created new opportunities for innovative telemedicine applications in health care. The COVID-19 pandemic has heightened the demand for contactless health care services, making SARS-CoV-2 polymerase chain reaction (PCR) testing a crucial component of pandemic containment.

**Objective:**

This feasibility study aimed to examine a comprehensive telemedicine approach for SARS-CoV-2 testing, focusing on the practicality, user satisfaction, and economic implications of self-sampling guided by a telemedicine platform.

**Methods:**

The study process involved shipping self-sampling kits, providing instructions for at-home sample collection, processing biomaterials (swabs and capillary blood), communicating test results, and providing interoperable data for clinical routine and research through a medical mobile app. A total of 100 individuals were randomly assigned to either the conventional health care professional (HCP)–performed SARS-CoV-2 testing group (conventional testing group, CG) or the telemedicine-guided SARS-CoV-2 self-sampling approach (telemedicine group, TG). Feasibility of the TG approach, user satisfaction, user-centered outcomes, and economic aspects were assessed and compared between the groups.

**Results:**

In the TG group, 47 out of 49 (95%) individuals received a self-sampling kit via mail, and 37 out of 49 (76%) individuals successfully returned at least one sample for diagnostics. SARS-CoV-2 PCR tests were conducted in 95% (35/37) of TG cases compared with 88% (44/50) in the CG. Users in the TG reported high satisfaction levels with ease of use (5.2/7), interface satisfaction (5.2/7), and usefulness (4.3/7). A microcosting model indicated a slightly higher cost for the TG approach than the CG approach. The TG demonstrated the potential to facilitate interoperable data transmission by providing anonymized, standardized datasets for extraction using Health Level 7-Fast Healthcare Interoperability Resources. This supports the national COVID-19 Data Exchange Platform and facilitates epidemiological evaluation based on the German COVID Consensus dataset.

**Conclusion:**

These preliminary findings suggest that a telemedicine-based approach to SARS-CoV-2 testing is feasible and could be integrated into existing hospital data infrastructures. This model has the potential for broader application in medical care, offering a scalable solution that could improve user satisfaction and treatment quality in the future.

## Introduction

COVID-19, caused by SARS-CoV-2, has globally brought health care systems to their limits [1]. An effective test strategy has proven to be a cornerstone in pandemic control and will be essential in future pandemics [2,3]. Self-performed rapid antigen tests have played an important role during the pandemic. However, in the health sector, especially when dealing with highly vulnerable patient groups, SARS-CoV-2 polymerase chain reaction (PCR) testing performed by health care professionals (HCPs) remains the gold standard because of its significantly higher sensitivity and specificity [4,5]. In addition, the SARS-CoV-2 serology allows for assessing past infections or the response to vaccinations [6].

Advances in technology have shaped the evolution of telemedicine over the last century. The universal availability of smartphones nowadays has opened up many new opportunities for the innovative use of telemedicine [7]. The COVID-19 pandemic has highlighted the demand and need for these contactless health care services [8]. However, suitable structures for comprehensive telemedical care still need to be improved in many public hospitals. Particularly, interoperability between different data processing systems and suitable platforms for patient communication is lacking [9].

At-home self-sampling has increasingly been used in many medical areas and can contribute to higher testing rates, particularly in stigmatized diseases, such as HIV or other sexually transmitted infections [10,11]. Our research group and others have demonstrated that people can reliably obtain swabs for SARS-CoV-2 PCR testing and self-collect capillary blood for SARS-CoV-2 serology [12-14]. These advancements paved the way for integrating self-sampling methods into telemedicine-based diagnostic platforms.

In this pilot study, we further developed a comprehensive telemedical SARS-CoV-2 PCR and serology testing platform during the early phase of the COVID-19 pandemic, including web-based data processing and result communication, and tested its feasibility. In total, 100 staff members at a German tertiary hospital were randomly assigned to either a group of conventional staff-guided, SARS-CoV-2 testing (conventional testing group, CG) or a telemedical SARS-CoV-2 diagnostic approach including self-sampling (telemedicine group, TG). We assessed and compared user satisfaction, patient-centered outcomes, and time and economic aspects in both groups. Interoperable data were used as the TG made anonymized, standardized datasets available for extraction using Health Level 7-Fast Healthcare Interoperability Resources (HL7-FHIR), contributing to the national COVID-19 Data Exchange Platform (CODEX) [15] and enabling epidemiological evaluation based on the German Corona Consensus (GECCO) [16] dataset.

## Methods

### Study Design

**Figure 1.**
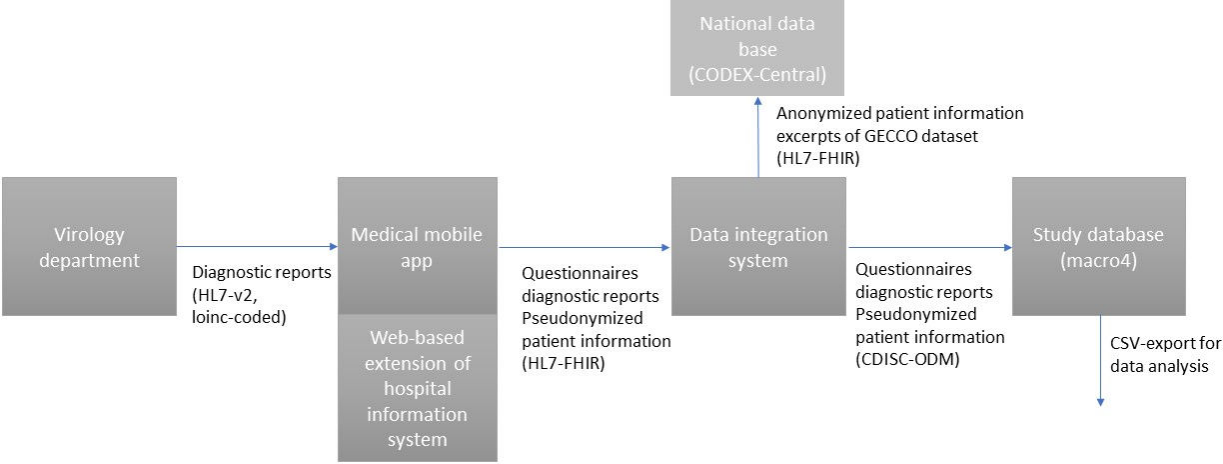
Data flow in a comprehensive telemedicine approach for SARS-CoV-2 self-sampling with 100 participants. CODEX: COVID-19 Data Exchange Platform; HL7-v2: Health Level 7, version 2; HL7-FHIR: Health Level 7-Fast Healthcare Interoperability Resources; LOINC: Logical Observation Identifiers Names and Codes; GECCO: German Corona Consensus dataset; CDISC-ODM: Clinical Data Interchange Standards Consortium-Operational Data Model; CSV: comma-separated values.

This monocentric, prospective, interventional, open-label, controlled, two-arm feasibility study was conducted at the university hospital Klinikum rechts der Isar (MRI) of the Technical University of Munich (TUM), Germany, in 2021. In total, 100 individuals were recruited from the medical staff in September and October based on specific inclusion criteria, including an indication for SARS-CoV-2 testing following the local government’s testing strategy (Bavarian Infection Protection Measures Ordinance) and the ability to download and use the user application of the software. Individuals with a known active SARS-CoV-2 infection were excluded. Randomization was performed in a 1:1 ratio to either the CG or TG for SARS-CoV-2 testing. e-Consent was obtained from all participants before randomization via a medical mobile app. The primary focus of this study was to establish a comprehensive infrastructure for the telemedicine concept and to test its feasibility. To achieve a complete telemedicical approach, we developed an easy-to-use user front-end application linked to a clinical information system and data integration center and improved interoperable data management (Figure 1). The CG underwent standard procedures for SARS-CoV-2 testing by HCPs at the study site.

To enable telemedicine care, the existing hospital information system (HIS) was expanded by incorporating additional functionalities through a web-based extension (HIS extension) for the study team and an additional mobile app version, which was available as a webpage and an iOS and Android app for the participants. The software served various functions, including study registration and randomization, assessment of indications for testing, printing shipping labels for self-sampling kits, report communication, and evaluation of user-reported parameters. Before study initiation, several internal test runs were performed.

### Registration and Randomization

The participants were informed about the study through flyers, by phone, or in person. Registration and informed consent were conducted at the hospital. The medical mobile app was downloaded by participants through a study-specific QR code with a deep link to the Android or Apple store (Figure S1 in Multimedia Appendix 1). A two-factor identification process was implemented to enhance the security of the registration process. After scanning the specific QR code, the second step of verification involved sending a letter with a specific QR code to the individual’s home address, which had to be scanned for further validation. Once the registration process was completed and the specific QR code was scanned, the study team performed a manual check to merge existing electronic patient files to grant study-specific access. A new study case was created, and after obtaining e-consent, randomization occurred at a 1:1 ratio into either the telemedicine or conventional testing group, using the Java function SecureRandom (algorithm: SHA1PRNG).

### Study Procedures for the Telemedicine Group

After randomization, participants were asked to complete a questionnaire in the medical mobile app. This questionnaire collected epidemiological data and clinical information regarding symptoms of SARS-CoV-2 infection, history of concomitant disease, and previous SARS-CoV-2 vaccination (Table S1 in Multimedia Appendix 1). Upon completion, the study team manually entered orders for the virology department, encompassing SARS-CoV-2 PCR testing and SARS-CoV-2 anti-N immunoglobulin G (IgG) and neutralizing antibodies testing. After creating a shipping label in the HIS extension, the self-sampling kit was dispatched using commercial post services (DHL, division of the German logistics company Deutsche Post DHL Group). To enhance user-friendliness, optimal user guidance was assured in the user front-end. A timeline was introduced to indicate the individual’s progress status in the study (Figure S2 in Multimedia Appendix 1). Push notifications and reminder emails were sent to provide further explanations regarding the next steps of the study procedure. The application used the DHL application programming interface (DHL API) for test kit tracking, and individuals could view the shipment status in their timeline. The self-sampling kit included testing materials, a prepaid shipping label for return, printed instructions regarding study procedures, and self-sampling (Figure S3 in Multimedia Appendix 1). For ease of use, pictograms (Figure S4 in Multimedia Appendix 1) and a video tutorial were specifically produced for this use case. Access to the video was provided via a QR code. After self-sampling, the samples were returned via DHL to the study center for diagnostic analysis. Test results for SARS-CoV-2 PCR and SARS-CoV-2 IgG and neutralizing antibodies were directly reported within the medical mobile app. Upon request, the participants could receive consultation about their test results via telephone.

### Study Procedures for the Conventional Testing Group

HCPs from the study team contacted individuals in the CG by phone. During this call, epidemiological data and symptoms suggestive of a SARS-CoV-2 infection were recorded based on the questionnaire used for the TG via the mobile app. Subsequently, an appointment was scheduled at the study center for HCP-performed nasopharyngeal swabs for SARS-CoV-2 PCR and a venous blood draw to determine SARS-CoV-2 IgG and neutralizing antibodies diagnostics. The results were communicated to the individuals via phone by an HCP. Patient-reported outcomes were gathered through the medical mobile app for comparative purposes with the TG.

### Duration for Shipment and Self-Sampling

The duration for shipment and self-sampling were recorded. In the TG, the DHL API monitored the time required to ship and return the test kits. Moreover, participants in the TG reported the time required to review the instructions and collect the samples via the mobile app. For the CG, the duration of HCP-conducted sampling, phone calls (initial questionnaire and result communication), and order entries were documented by the study team in the HIS extension.

### Cross-System Data Acquisition and Communication

Standard-based data communication was used among all study components, including the medical mobile app, HIS, the data integration center, and the study database (Figure 1). Reports from the virology department containing five parameters (method, analyte, material, quality or quantity, and measurement parameter) were transmitted using HL7-v2 and encoded with logical observation identifier names and codes. The time and location of sampling (for example, nasopharyngeal or midturbinate) were added manually. SARS-CoV-2 results were imported into the medical mobile app to be viewed and downloaded in portable document format.

Virology results were extracted via pull from the HIS extension using Fast Healthcare Interoperability Resources (HL7-FHIR) and transferred along with the questionnaire data collected in the mobile app into an electronic case report form through the local data integration system of the Medical Informatics Initiative Germany and data integration for future medicine (DIFUTURE). Pseudonymized data for all study participants were fed into the study-specific electronic data capture system (Macro4) using the Clinical Data Interchange Standards Consortium-Operational Data Model, as used by the Münchner Studienzentrum (Munich study center) of TUM for study data evaluation. Information on personal data (age, sex, contact details, and insurance information) and COVID-19–related details (symptoms, SARS-CoV-2 transmission risks, previous SARS-CoV-2 vaccination, and risk factors according to the GECCO dataset) were collected in both groups. To allow research use on the German national level, anonymized and standardized datasets from the TG regarding SARS-Cov-2 were provided to CODEX [15] for epidemiological evaluation following the GECCO [16] dataset in HL7 FHIR.

### Economic Model Evaluation

Resource utilization and time costs were assessed by expert ratings, questionnaire questions, and information extracted from the study database. A simple model for a microcosting approach was developed to analyze the differences for various scenarios and assumptions. The model was implemented in R (version 4.2.1, open-source software) and RStudio (Posit PBC).

### User Satisfaction

To assess user-centered outcome parameters, standardized questionnaires were administered to both groups in the user front-end of the medical mobile app following the communication of PCR and serology results at the end of the study journey. User satisfaction was gauged using a Likert scale as part of a standardized questionnaire. Ergonomic and usability data specific to the telemedicine group were gathered using the mHealth App Usability Questionnaire (MAUQ) score [17] and an adapted National Aeronautics and Space Administration (NASA) task load index score [18]. In addition, the instructions for self-sampling and the general acceptance of the telemedicine approach were evaluated.

### Material and Diagnostic Methods

The self-sampling kit was shipped through DHL, using packaging compliant with the United Nations’ recommendation for dangerous goods (UN 3373). The kit included an oropharyngeal swab (REST Clinical Virus Transport Medium [CTM] swab, Rapid & Easy System Technology, Noble Biosciences Inc) and a midturbinate swab (FLOWSwab) [19], both for SARS-CoV-2 PCR, as well as material for capillary blood sampling (1.2 mL) for serology (Thermacor Micro Vial, MedDX Solutions), Secondary 95 kPa Pouch (MedDX Solutions), Microtainer and Microtainer lancet (Becton Dickinson) [14]. Virological diagnostics were performed by the Institute of Virology, TUM, adhering to established standard operating procedures. iFlash-SARS-CoV-2 IgG assay (YHLO) detected antibodies directed against an antigen mixture, mainly N-antigen. The iFlash-2019-nCoV neutralizing antibody assay quantitated neutralizing antibodies against the receptor-binding domain of the spike antigen. Both tests were run on an iFLASH 1800 analyzer. SARS-CoV-2 RNA was detected qualitatively in swabs with the Cobas SARS-CoV-2 assay (Roche, Mannheim, Germany) on the Cobas 6800 system.

The manuscript was prepared in accordance with the iCHECK-DH: Guidelines and Checklist for the Reporting on Digital Health Implementations [20].

### Sample Size Calculation and Statistical Methods

No formal sample size estimation was conducted for this feasibility study. However, we hypothesized that 100 participants would be sufficient to demonstrate the feasibility of the procedures. A total of 50 participants in each group sufficed for constructing two-sided 95% CIs with a reasonable width. The full analysis set comprised all participants who were included, randomized, and tested for SARS-CoV-2 in this study. Missing values were not imputed in the analysis. Analyses were performed using SAS version 9.4 (SAS Institute Inc), R version 4.2.3 (R Foundation for Statistical Computing), and RStudio version 2023.03.0 (Posit Software, PBC).

### Ethical Considerations

This study was approved by the Ethics Committee at TUM, School of Medicine, University Hospital Klinikum rechts der Isar, Munich, Germany (approval number 267/21 S) and was conducted in accordance with the principles of the Declaration of Helsinki. These principles encompass ethics committee procedures, patient education, informed consent, adherence to the protocol, administrative documentation, data collection, patient records (source documents), and the secure storage and retention of documents. All investigators and staff involved in the study were fully informed of the protocol, study procedures, and their respective roles.

The study was registered with the national registry Deutsches Register Klinischer Studien (DRKS) under the registry number DRKS00027093. e-Consent was obtained from all participants via a medical mobile app before randomization. Study data were collected in a pseudonymized form to ensure the privacy and confidentiality of all participants. No compensation was provided to the participants in this study.

## Results

### Overview

In September and October 2021, 100 staff members of the university hospital MRI of TUM, who had an indication for SARS-CoV-2 testing but no known active SARS-CoV-2 infection, were recruited to participate in the study. They were randomly assigned in a 1:1 ratio into TG or CG for SARS-CoV-2 testing.

Demographic characteristics were balanced between the TG and CG. Individuals had a mean age of 38 (SD 11.1) years in the TG and 36 (SD 11.8) years in the CG. Women outnumbered men, comprising 69.4% (34/49) in the TG and 68.6% (39/51) in the CG.

**Figure 2.**
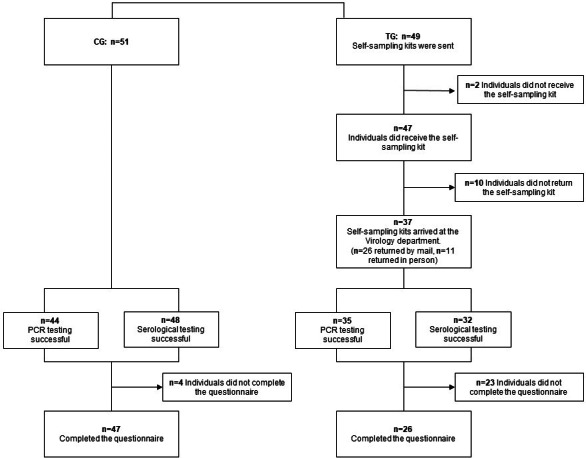
Study procedures in the conventional testing group (CG) and the telemedicine group (TG) of a comprehensive telemedicine approach for SARS-CoV-2 testing. PCR: polymerase chain reaction.

In the TG, all 49 self-sampling kits were dispatched and received by 47 individuals (Figure 2). In 2 instances, self-sampling kits were not delivered on time owing to the recipients’ inability to accept the packages for various reasons. A total of 37 of the 49 individuals (76%) returned their self-sampling kit with at least one sample to the laboratory. PCR tests for SARS-CoV-2 could be performed on 35/37 (95%) of the TG samples and 44/51 (88%) of the CG samples. All SARS-CoV-2 PCRs from oropharyngeal and midturbinate swabs yielded negative results in both groups. Overall, 34 of 49 individuals in the TG (69.4%) returned a blood sample, of which 32 (94.1%) had sufficient quality and quantity to conduct the serology. Serology testing was conducted for 48/51 individuals (94.1%) in the CG of which all were successfull.

The average shipping time for self-sampling kits was 42 hours [minimum 16 h; maximum 241 h]. Upon arrival, it took an average of 201 (SD 190) hours (range 40-788) to return them via mail. The average duration from reshipping to diagnostic arrival was 55 (SD 32) hours (3-112). Information on the time needed for self-sampling was available from 25 individuals (51%) and estimated at 16.7 minutes on average (95% CI 12.9-20.5). Professional testing of the TG participants took an average of 26.2 minutes (95% CI 19.4-33.0). We observed a trend toward a reduced testing duration in the TG. However, this did not reach statistical significance.

Communication of testing results via the medical mobile app was successful in all TG participants. In the CG, although all individuals were accessible via telephone, repeated attempts were required for some owing to initial nonresponse. Information on epidemiological data, SARS-CoV-2 symptoms, previous SARS-CoV-2 infection, SARS-CoV-2 vaccination, SARS-CoV-2 risk contacts, and concomitant disease history (Table S1 in Multimedia Appendix 1) was obtained in 26/49 (53%) individuals in the TG using the mobile app and 51/51 (100%) in the CG via a telephone call.

### User Satisfaction

A total of 26/49 individuals (53.1%) completed the questionnaires in the TG, compared with 47/51 (92%) in the CG.

**Figure 3.**
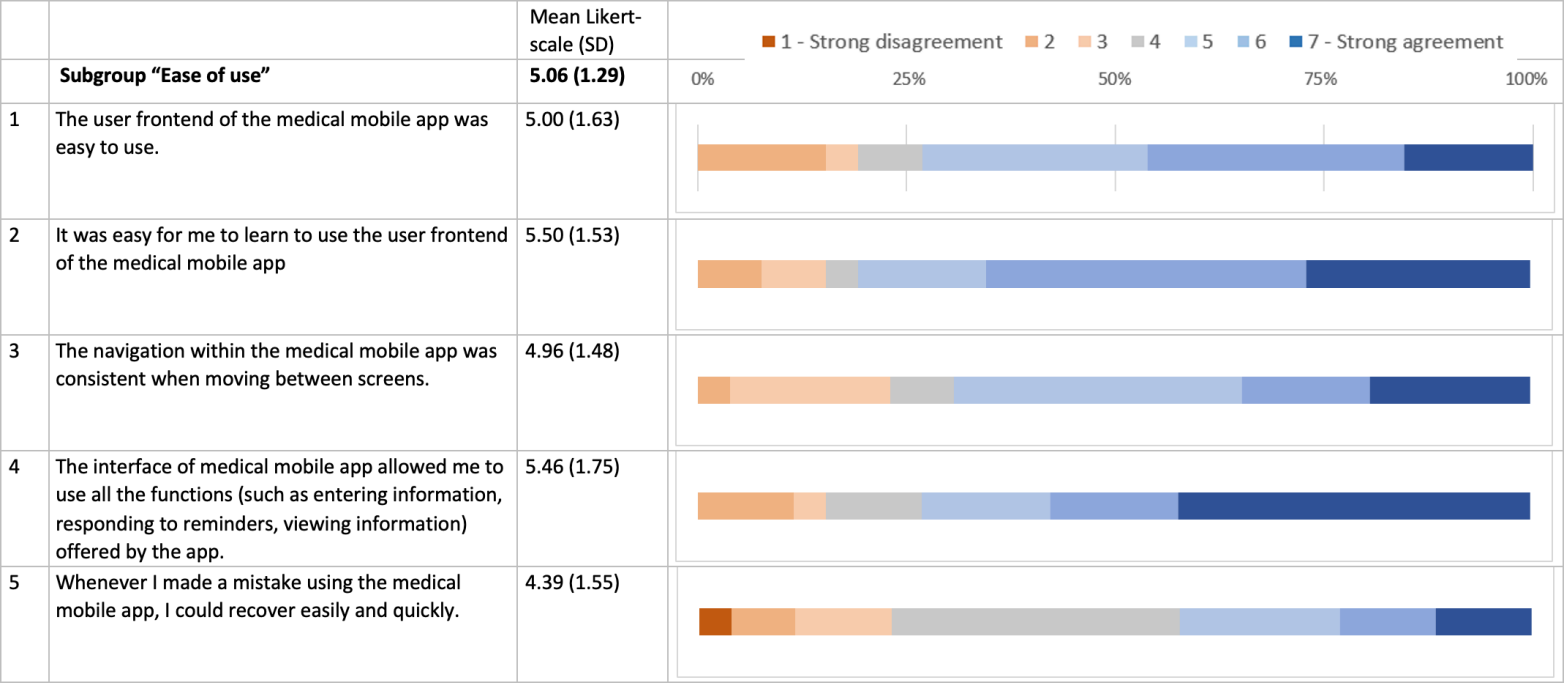
An overview of the mHealth App Usability Questionnaire (MAUQ) concerning satisfaction with telemedicine for the telemedicine group (TG) in a comprehensive approach for SARS-CoV-2 testing. The bars show the fractions of agreement for the MAUQ score in the TG for the subcategory “ease of use” (questions 1‐5). A total of 26/49 (53%) individuals completed the questionnaires. MAUQ (TG only) was measured on a Likert scale of 1‐7 (1=strong disagreement, 7=strong agreement).

**Figure 4.**
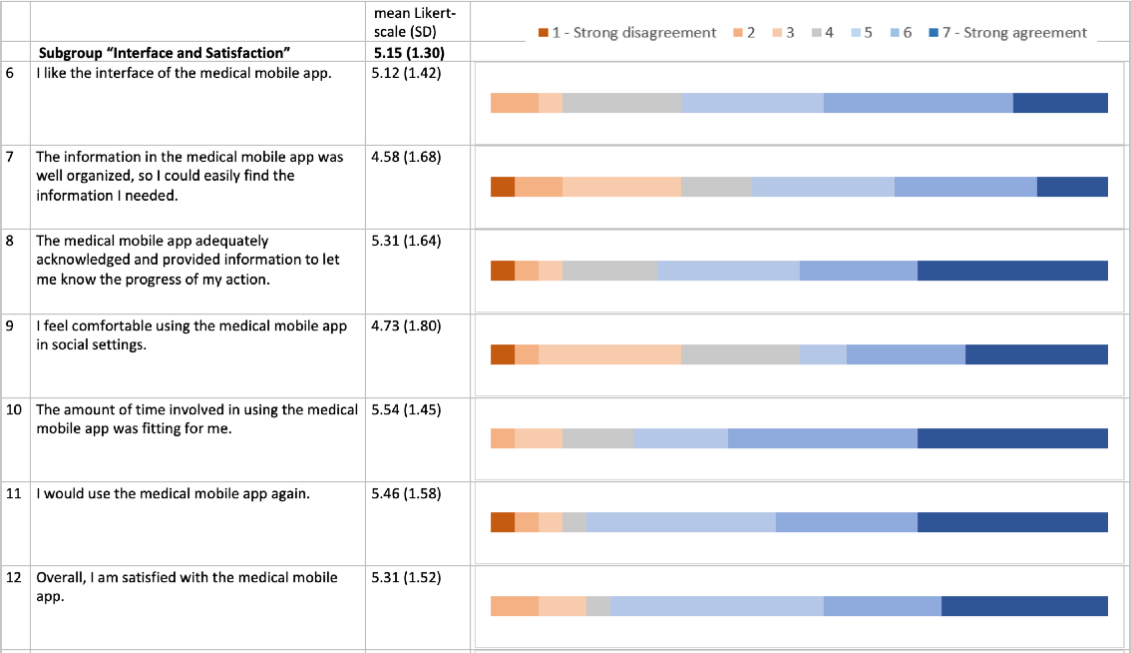
An overview of the mHealth App Usability Questionnaire (MAUQ) concerning satisfaction with telemedicine for the telemedicine group (TG) in a comprehensive approach for SARS-CoV-2 testing. The bars show the fractions of agreement for the MAUQ score in the TG for the subcategory “interface and satisfaction” (questions 6‐12). A total of 26/49 (53%) individuals completed the questionnaires. MAUQ (TG only) was measured on a Likert scale of 1‐7 (1=strong disagreement, 7=strong agreement).

**Figure 5.**
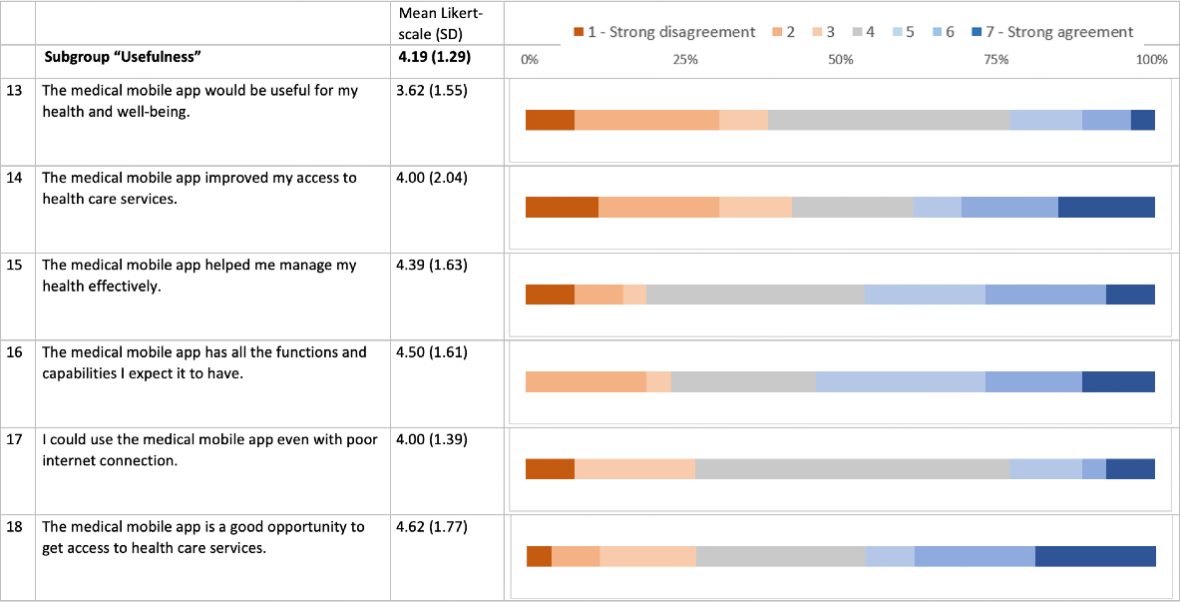
An overview of the mHealth App Usability Questionnaire (MAUQ) concerning satisfaction with telemedicine for the telemedicine group (TG) in a comprehensive approach for SARS-CoV-2 testing. The bars show the fractions of agreement for the MAUQ score in the TG for the subcategory “usefulness” (questions 13‐18). A total of 26/49 (53%) individuals completed the questionnaires. MAUQ (TG only) was measured on a Likert scale of 1‐7 (1=strong disagreement, 7=strong agreement).

TG individuals expressed high satisfaction in the MAUQ, which assesses the usability of mobile health applications using a Likert scale from 1 to 7 with higher values representing “strong agreement.” The mean (SD) scores for the MAUQ subcategories were as follows: ease of use at 5.1 (SD 1.3), interface and satisfaction at 5.2 (SD 1.3), and perceived usefulness at 4.2 (SD 1.3; Figures 3-5). The results of the individual questions are graphically depicted in Figures 3-5. However, it is important to note that these detailed scores are based on a relatively small sample size, which may limit the generalizability of the specific findings.

In the TG, individuals demonstrated high satisfaction with the medical mobile app for test result communication, evidenced by mean Likert scale scores mainly above 4.5 on a 6-point scale (Table 1). Accessing and understanding the SARS-CoV-2 PCR and serology results was straightforward, suggesting a preference for this digital communication over traditional phone methods. Furthermore, the concept of self-sampling was positively received, coupled with telemedicine communication for future use. Regarding the instructional videos for throat or nasal swabs and capillary blood samples, their comprehensibility and level of detail were well received, with an average score of 5.1 on a 6-point Likert scale (Table S2 in Multimedia Appendix 1). Individuals in the TG generally expressed high confidence and satisfaction with performing self-sampling and packaging. Owing to initial technical issues, return shipping received a relatively low rating of 2.2 out of 6 (Table S3 in Multimedia Appendix 1).

**Table 1.** Agreement to testing result communication (only telemedicine group, TG) in a telemedicine approach for SARS-CoV-2 testing, measured on a Likert scale from 1-6 (1=strong disagreement, 6=strong agreement). Out of 49 individuals in the TG, 25 returned their questionnaires.

	English translation of the questions	Score, mean (SD)
1	The communication of the test result via the user frontend of the medical mobile app is convenient.	5.36 (0.91)
2	Finding the test result in the medical mobile app is easy.	5.20 (1.29)
3	I prefer the communication of the results via the medical mobile app to communication via phone.	4.48 (1.76)
4	I understood the test result of the SARS-CoV-2 PCR^a^.	5.52 (1.23)
5	I understood the test result of the SARS-CoV-2 serology.	4.40 (1.89)
6	I understand the test result and its subsequent consequences.	5.16 (1.43)
7	The written report of the test results available in the medical mobile app allows me to consider the consequences or the questions regarding the test result.	4.68 (1.62)
8	The explanations provided along with the test result in the medical mobile app are sufficient, and I have no further questions.	4.44 (.61)
9	I did not feel left alone with my test result not being able to ask individual questions.	4.48 (1.56)
10	I did not have concerns regarding data protection when my test result is communicated via the medical mobile app.	4.88 (1.33)
11	Overall, I liked the approach of the self-sampling for SARS-CoV-2 testing and the telemedicine communication of the test result.	4.72 (1.40)
12	I will be able to manage the self-collection with shipped test kits as for SARS-CoV-2 testing in a different scenario similar to this study.	5.01 (1.12)
13	In principle, I would perform self-sampling again.	4.76 (1.42)

a PCR: polymerase chain reaction.

The TG and the CG expressed similar levels of interest and comfort in self-sampling at home, perceiving it as flexible and time-efficient (Table S4 in Multimedia Appendix 1).

Individuals in the CG generally reported high satisfaction with the efficiency and ease of scheduling and experiencing HCP-guided SARS-CoV-2 sampling, with most aspects scoring above 4.5 on a 6-point Likert scale, although the comfort of the nasopharyngeal sampling process was rated lower at 3 (Table S5 in Multimedia Appendix 1). Despite being content with phone communication for test results, they preferred telemedicine, demonstrating interest in self-collection and digital communication of results implemented in the interventional group (TGTable S6 in Multimedia Appendix 1).

### Economical Evaluation

A microcosting model was developed to compare the costs connected to the two different approaches. Details about the model and its assumptions are provided in Multimedia Appendix 2. Three scenarios with different numbers of patients (400, 2000, and 100,000) were evaluated. In all scenarios, the telemedicine approach led to higher costs than the conventional testing approach, with fewer individuals leading to higher cost differences. This association can be explained by the fixed costs connected to the telemedicine approach. If one-time fixed costs (such as app development costs) are disregarded, the cost difference ranges from about €6 (US $6.96; 400 individuals scenario) to about €3 (US $3.48; 100,000 individuals scenario). If one-time fixed costs are considered, the cost difference ranges from about €54 (US $62.64; 400 individuals scenario) to about €3 (US $6.96; 100,000 individuals scenario).

## Discussion

### Principal Findings

This monocentric, prospective, controlled study demonstrated the feasibility of a telemedicine-based SARS-CoV-2 testing versus conventional HCP-guided testing.

We provided insights into a mobile app-based self-sampling strategy with a user-friendly front-end and contactless, digital result communication. We evaluated user satisfaction, time efficiency, and economic implications of integrating at-home self-sampling and digital health solutions into clinical diagnostics. Furthermore, we established the feasibility of contributing our study data to a national research platform for further analysis.

In the TG, self-sampling kits were dispatched to the participants, allowing them to collect oropharyngeal swabs and capillary blood at home. The collected samples were returned to the laboratory via post service, and testing results were communicated to the user in the front-end application. In general, the telemedicine approach has proven its feasibility.

Dispatching the self-sampling kits via DHL after registration and checking the testing indication with the symptom questionnaire was successful. However, only 37 of 47 shipped kits were returned to the laboratory. We assume that this relatively low return rate can be ascribed to a lack of motivation to complete the optional diagnostics rather than a lack of understanding or difficulty in carrying out the procedure. This is supported by the fact that individuals in the TG generally expressed confidence and satisfaction in self-sampling, packaging, and shipping the samples. The successful usability of 95% of swabs and 94% of blood samples for diagnostics demonstrates that lay persons can effectively perform self-sampling with thorough instruction. However, as the study participants were medical personnel, this might have slightly enhanced the overall success rate, although the findings are still broadly encouraging [12,13]. Notably, the study highlights significant user acceptance and satisfaction with the mobile health app among the TG participants, as indicated by high scores in the MAUQ and a preference for digital communication over traditional phone methods. Users found navigating and interpreting SARS-CoV-2 PCR and serology results intuitive, appreciating the straightforward approach. Integrating self-sampling with telemedicine was well received, with participants from both TG and CG valuing the convenience and time-saving aspects of self-sampling.

According to microcosting model analysis, the telemedicine approach appears costlier than conventional testing in all scenarios (involving 400, 2000, and 100,000 patients). Notably, the cost disparities are more pronounced in smaller patient groups, primarily owing to fixed costs associated with the telemedicine method. The underlying assumptions and their inherent uncertainties should be considered when interpreting these findings. While it may seem improbable that the telemedicine approach offers a substantial cost advantage over the conventional approach guided by HCPs, other important factors must be considered with caution. This method of self-collection reduces the utilization of resources, such as personal protective equipment at testing centers and also eliminates the need for HCP personnel during the swab collection process.

Furthermore, export of structured datasets (based on the GECCO dataset) to the national data exchange platform (CODEX) facilitates national-level analyses for research and future pandemic management.

This study has some limitations. The participants were relatively young, demonstrating a higher affinity and preference for digital solutions than other patient groups. In addition, the high reliability of the execution and the confidence and comfort in self-sampling could be partly due to the medical background of the staff. Therefore, the results should be verified in other patient cohorts. In the TG, unfortunately, only 26/49 individuals (53.1%) completed their questionnaires, raising concerns about a potential nonresponse bias. The low response rate could be explained by the study being conducted at the peak of the pandemic when hospital employees were subjected to considerable stress. In addition, the small sample size limits the precision of our findings, and the detailed results should be viewed as exploratory. Further validation in larger, more diverse populations is therefore needed.

In general, telemedicine approaches will play a more significant role in the future in all aspects of medicine, not just in pandemic situations where minimizing physical contact is essential.

Telemedicine approaches have been established in several medical settings, such as cardiology, using tele-electrocardiograms (ECGs) and remote monitoring of cardiac diseases through wearables and tele-ECGs [21,22]. Specialties, such as dermatology, could be of particular interest, as sharing photos of skin conditions enables immediate consultation [23]. The application scope of telemedicine has been significantly broadened by at-home self-sampling of biomaterials. Particularly in stigmatizing infectious pathogens such as HIV, human papillomavirus, or other sexually transmitted infections, the combination of telemedicine with self-sampling may increase testing willingness and, consequently, the testing rate [10,11,24]. Remarkably, for blood collection, future systems designed for self-sampling by laypeople could enhance comfort [25,26]. In conclusion, a telemedicine approach incorporating at-home self-sampling for SARS-CoV-2 PCR and serology testing proved feasible and was met with high user satisfaction. The four major strengths of this approach include high availability, potential for reduced contact in infectious disease scenarios, conservation of HCP capacities, and the provision of standardized datasets for clinical research and pandemic management.

### Conclusion

A telemedicine approach incorporating at-home self-sampling for SARS-CoV-2 PCR and serology testing proved feasible and was met with high user satisfaction. The four major strengths of this approach include high availability, potential for reduced contact in infectious disease scenarios, conservation of HCP capacities, and the provision of standardized datasets for clinical research and pandemic management.

## Supplementary material

10.2196/57608 Multimedia Appendix 1 Supplementary material on clinical data, user satisfaction, and self-sampling manuals.

10.2196/57608 Multimedia Appendix 2 Economical evaluation.

## Abbreviations

API: application programming interface
CG: conventional testing group
CODEX: COVID-19 Data Exchange Platform
CSV: comma-separated values
DIFUTURE: Data Integration for Future Medicine
DIS: data integration center
ECG: electrocardiogram
GECCO: German Corona Consensus
HCP: health care professional
HIS: hospital information system
HL7-FHIR: Health Level 7-Fast Healthcare Interoperability Resources
IgG: immunoglobulin G
LOINC: Logical Observation Identifiers Names and Codes
MAUQ: mHealth App Usability Questionnaire
MRI: Klinikum rechts der Isar
PCR: polymerase chain reaction
TG: telemedicine group
TUM: Technical University of Munich
