# Supplementary material for: Implementation and User Satisfaction of a Comprehensive Telemedicine Approach for SARS-CoV-2 Self-Sampling: Monocentric, Prospective, Interventional, Open-Label, Controlled, Two-Arm Feasibility Study
Source: JMIR Form Res. 2024 Dec 11;8:e57608. doi: 10.2196/57608 (PMC11655044; doi:10.2196/57608)
Supplement: Multimedia Appendix 1 [file formative-v8-e57608-s001.docx]

**Supplementary Figures and Tables**

Supplementary Table 1. Questionnaire 1: Specific items on epidemiological data, SARS-CoV-2 symptoms, previous SARS-CoV-2 infection, SARS-CoV-2 vaccination, SARS-CoV-2 risk contacts and concomitant diseases history, as asked in both groups of a comprehensive telemedicine approach for SARS-CoV-2 self-sampling.

| Category | Specific information |
| --- | --- |
| Epidemiological data | name, gender, date of birth, age, address, email address, insurance status |
| SARS-CoV-2 symptoms | Did you recognize one of the following symptoms within the last few weeks? Cough, fever, shortness of breath, headache, diarrhoea, abdominal pain, nausea, loss of taste/smell. |
| Previous SARS-CoV-2 infection | Have you tested positive for SARS-CoV-2 before within the last four weeks? |
| SARS-CoV-2 vaccination | Have you been vaccinated against SARS-CoV-2? If yes, how many SARS-CoV-2 vaccinations did you receive? What was the date of your last SARS-CoV-2 vaccination? |
| SARS-CoV-2 risk contacts | Did you have contact with a confirmed case of SARS-CoV-2 within the last 14 days? Was your contact in private or work setting? Did you travel to an area of risk within the last 14 days? |
| concomitant diseases history | Do you have one of the following pre-existing medical conditions? (Diabetes mellitus, COPD/Asthma, immunosuppression, cardiovascular diseases) |

Supplementary Table 2. Evaluation of video manual (only telemedicine group, TG) on a Likert-scale from 1-6 (1=strong disagreement, 6=strong agreement). Out of 49 individuals 26 questionnaires were returned. SD = standard deviation.

|  | English translation of the questions | mean Likert-scale (SD) |
| --- | --- | --- |
| 1 | The video manual for the throat/nasal swabs was easy to comprehend. | 5.42 (1.34) |
| 2 | The video manual for the throat/nasal swabs is detailed enough. | 5.00 (1.39) |
| 3 | The video manual for the capillary blood samples was easy to comprehend. | 5.42 (1.07) |
| 4 | The video manual for the capillary blood samples is detailed enough. | 5.08 (1.35) |
| 5 | The video manual for packing the samples was easy to comprehend. | 5.15 (1.12) |
| 6 | The video manual for packing the samples is detailed enough. | 4.73 (1.51) |

Supplementary Table 3. Evaluation of self-sampling (only telemedicine group) on a Likert-scale 1-6 (1=strong disagreement, 6=strong agreement). Out of 49 individuals in the telemedicine group 25 returned their questionnaires. SD = standard deviation.

|  | English translation of the questions | Mean Likert-scale (SD) |
| --- | --- | --- |
| 1 | Performing the self-collection of swabs for SARS-CoV-2 testing was simple and successful from my point of view. | 5.04 (1.43) |
| 2 | I am happy with the self-collection procedure of the swab for SARS-CoV-2 testing. | 5.04 (1.34) |
| 3 | Self-collection of the swab for SARS-CoV-2 testing requires no mental effort because the task is complex. Self-collection of the SARS-CoV-2 swab is technically easy. | 3.72 (1.06) |
| 4 | I felt certain while performing the self-collection procedure of the SARS-CoV-2 swab. | 5.00 (1.19) |
| 5 | Performing the self-collection of the swab for SARS-CoV-2 testing did not frustrate me. | 5.44 (1.12) |
| 6 | Performance of the midturbinate swab was less convenient than the nasopharyngeal swab. | 3.48 (1.85) |
| 7 | Performance of the midturbinate swab was more difficult than the nasopharyngeal swab. | 2.92 (1.63) |
| 8 | I received the self-sampling kit within an appropriate time span. | 5.64 (0.70) |
| 9 | Return shipping of the self-sampling kit was practicable. | 2.16 (1.80) |
| 10 | Manageability of the packaging was practicable. | 5.01 (1.26) |
| 11 | Packaging of the swab tubes was technically easy. | 5.48 (0.92) |
| 12 | The packaging of the tubes took me less than 15 minutes. | 5.2 (1.56) |

Supplementary Table 4. Evaluation of self-sampling (both groups) on a Likert-scale from 1-6 (1=strong disagreement, 6=strong agreement). SD = standard deviation. No statistically significant differences were detected for all items.

|  | English translation of the questions | mean Likert-scale (SD) TG | mean Likert-scale (SD) CG |
| --- | --- | --- | --- |
| 1 | SARS-CoV-2 self-sampling at home are of great interest to me, compared to health care professional-guided sampling. | 4.32 (1.73) | 4.00 (1.53) |
| 2 | I would prefer SARS-CoV-2 self-sampling at home and shipment of a test kit, compared to an appointment with medical staff and health care professional-guided sampling. | 4.00 (1.87) | 3.77 (1.54) |
| 3 | SARS-CoV-2 self-sampling allow higher flexibility regarding time, compared to health care professional-guided sampling at a test center. | 4.68 (1.46) | 4.79 (1.22) |
| 4 | SARS-CoV-2 self-sampling is less time-consuming than collection of the swab by a health-care professional at a test center. | 4.44 (1.83) | 4.75 (1.51) |
| 5 | The self-collection of swabs for SARS-CoV-2 testing seems to be more secure to me in terms of the infection risk (considering the exposure on the way to a test center and at the test center), compared with the collection of the swab by a health-care professional at a test center. | 3.68 (1.73) | 3.43 (1.44) |
| 6 | The self-collection of swabs for SARS-CoV-2 testing is less comfortable than the collection of swabs by a health-care professional at a test center. | 3.92 (1.50) | 3.68 (1.56) |
| 7 | Capillary blood self-collection is less comfortable than getting blood drawn by a health-care professional. | 2.84 (1.75) | 2.51 (1.30) |
| 8 | Performance of serology is of extra profit for me. | 4.80 (1.68) | 4.96 (1.22) |

Supplementary Table 5. Evaluation of sampling through healthcare professionals (only SOC group) on a Likert-scale 1-6 (1=strong disagreement, 6=strong agreement). Out of 51 subjects in the SOC group 47 returned their questionnaire. SD = standard deviation.

|  | English translation of the questions | Mean Likert-scale (SD) |
| --- | --- | --- |
| 1 | I got a prompt appointment via telephone health-care professional-guided SARS-CoV-2 sampling at a test center. | 4.47 (1.54) |
| 2 | I did not have to wait long for my appointment for health-care professional-guided SARS-CoV-2 sampling. | 5.38 (1.03) |
| 3 | The appointment for health-care professional-guided SARS-CoV-2 sampling did not take much more time than expected. | 5.19 (1.28) |
| 4 | Health-care professional-guided SARS-CoV-2 sampling happened without any problems. | 5.79 (0.62) |
| 5 | I am content with the procedure of my SARS-CoV-2 sampling. | 5.77 (0.76) |
| 6 | The procedure of nasopharyngeal SARS-CoV-2 sampling was not unpleasant. | 3.00 (1.66) |

Supplementary Table 6. Result communication (only SOC group) on a Likert-scale from 1-6 (1=strong disagreement, 6=strong agreement). Out of 51 subjects in the SOC group 47 returned their questionnaires. SD = standard deviation.

|  | English translation of the questions | Mean Likert-scale (SD) |
| --- | --- | --- |
| 1 | The communication of the test result via call was seamless. | 3.64 (1.80) |
| 2 | I received the call regarding my test result without delay. | 4.53 (1.37) |
| 3 | I understood the test result of the SARS-CoV-2 PCR. | 5.43 (1.10) |
| 4 | I understood the test result of the SARS-CoV-2 serology. | 5.04 (1.59) |
| 5 | I understood the test report communicated over call and its subsequent consequences. | 4.23 (1.72) |
| 6 | I wish to have received a written report for considering the result and its consequences and the questions. | 4.30 (1.57) |
| 7 | I feel safe with the test result provided by phone. | 5.23 (1.17) |
| 8 | I would like the approach of the self-collection of the swab for SARS-CoV-2 testing and the digital communication of the test result, as performed in the other study group. | 4.36 (1.39) |
| 9 | I think I would be able to manage the self-collection of the swab for SARS-CoV-2 testing in a different scenario similar to the approach of the other group in this study. | 4.98 (1.32) |
| 10 | I would choose an overall concept for self-sampling. | 4.51 (1.43) |

Supplementary Figure 1. QR code for download of the medical application used for a comprehensive approach of SARS-CoV-2 self-sampling.


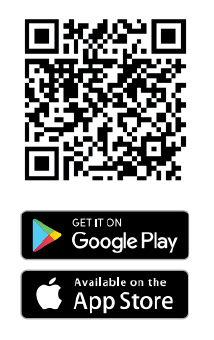


Supplementary Figure 2. User progress status timeline in the medical application for a comprehensive approach of SARS-CoV-2 self-sampling.


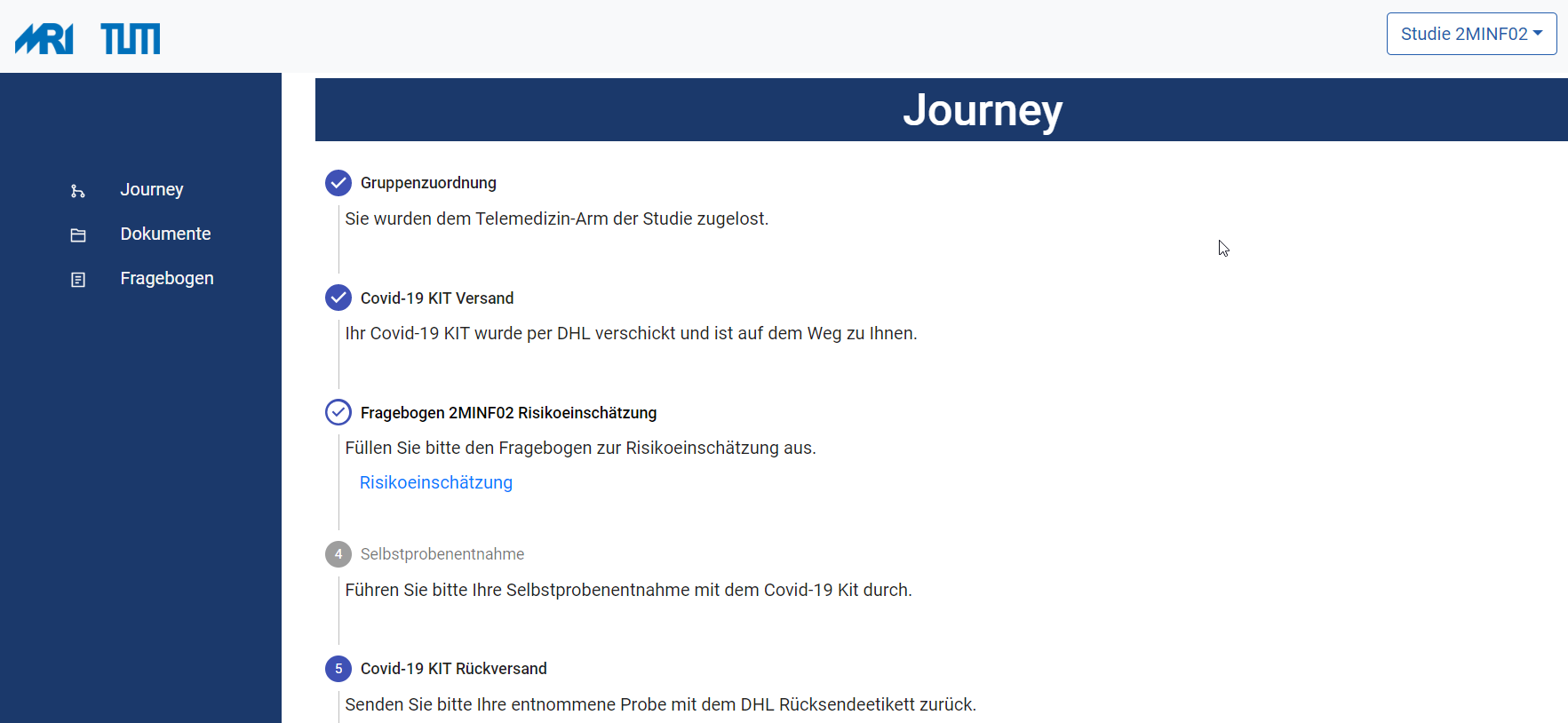


Supplementary Figure 3. Self-sampling kit with all components for SARS-CoV-2 self-sampling.


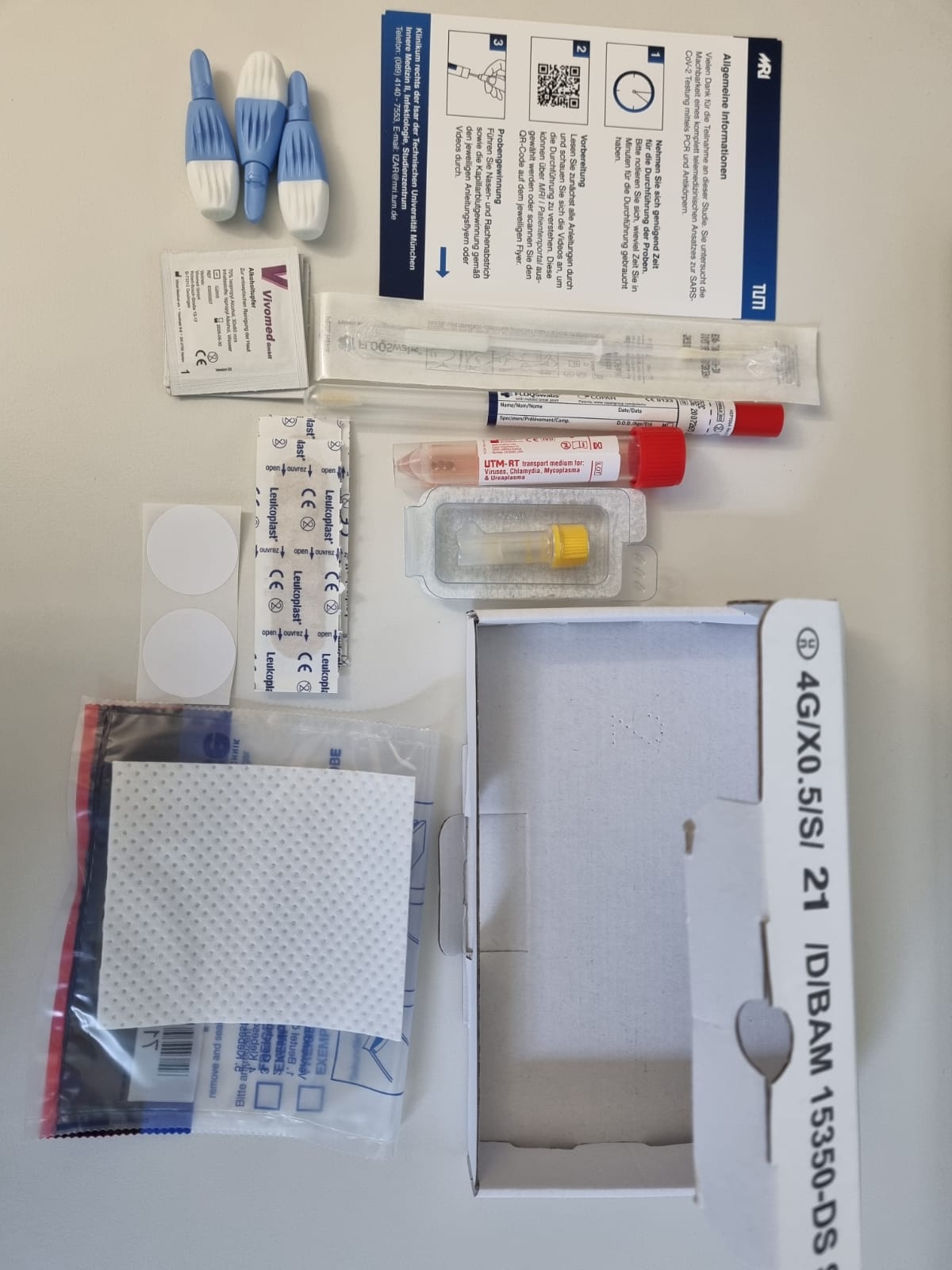


Supplementary Figure 4. Manuals for SARS-CoV-2 self-sampling with pictograms as used in a comprehensive telemedicine approach


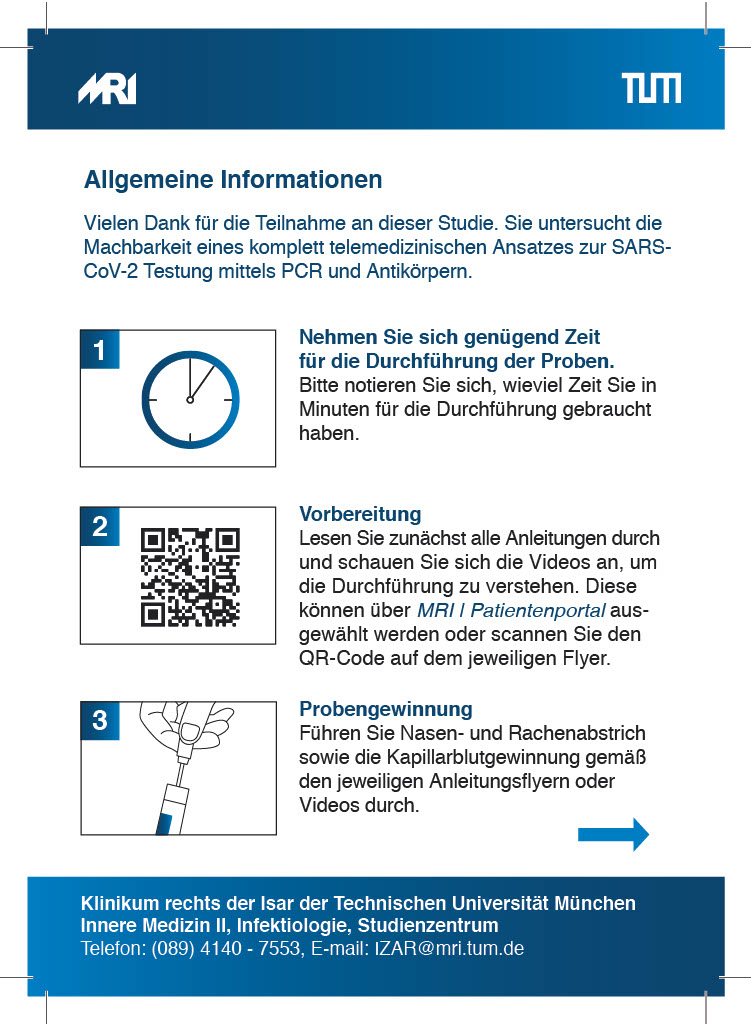

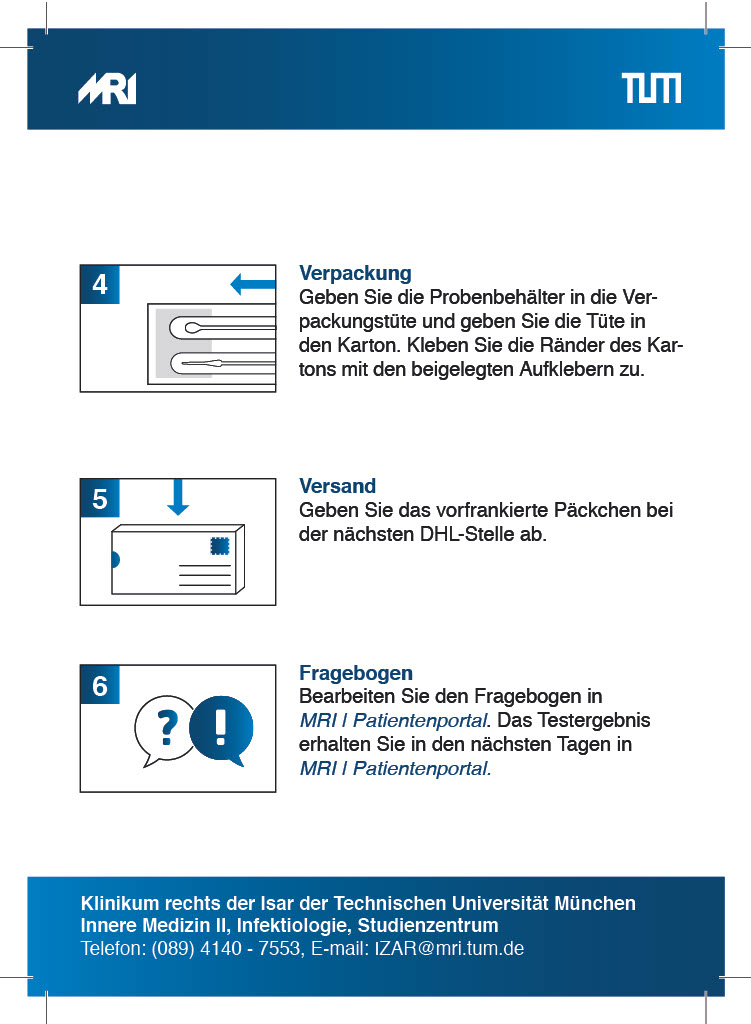

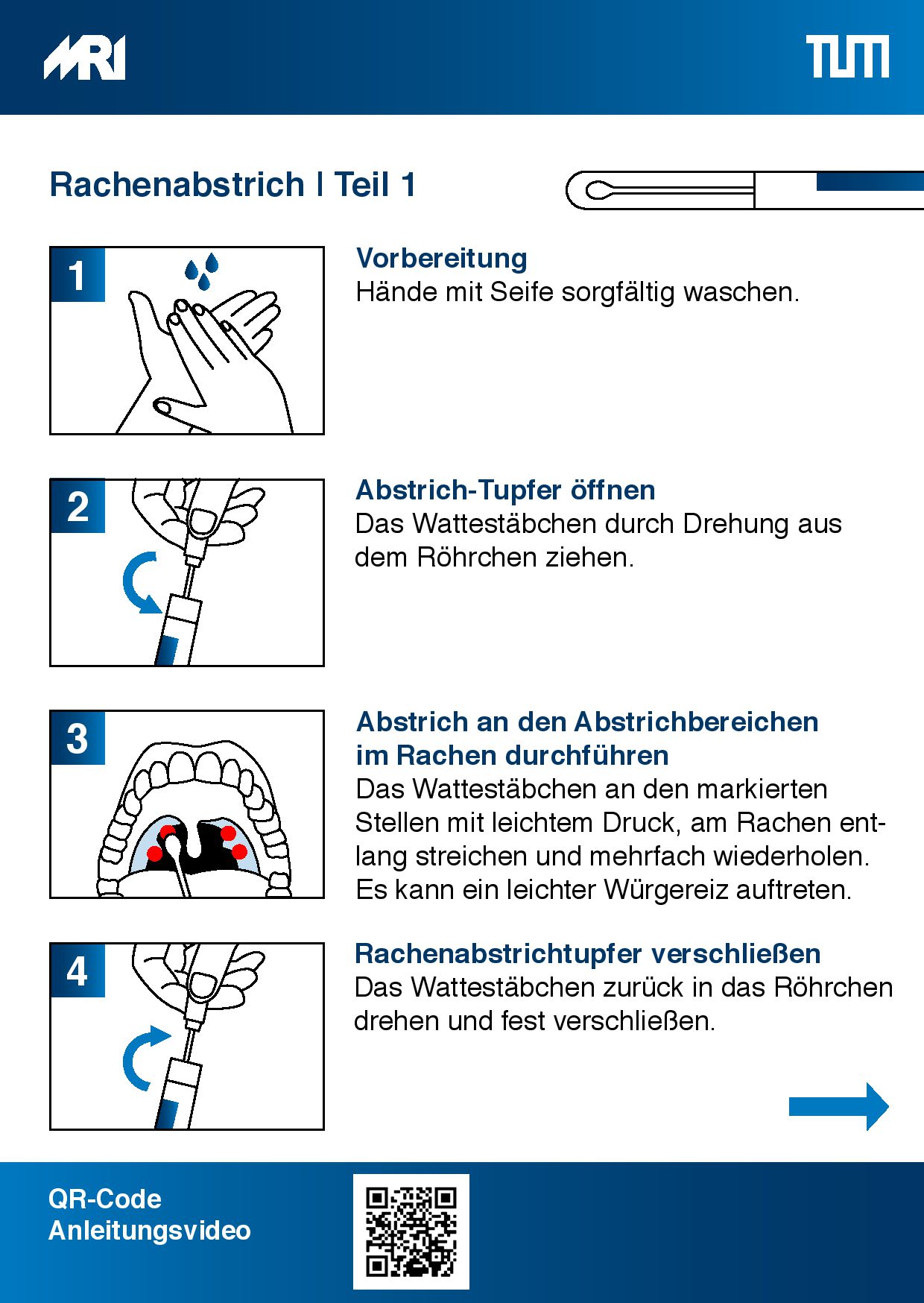

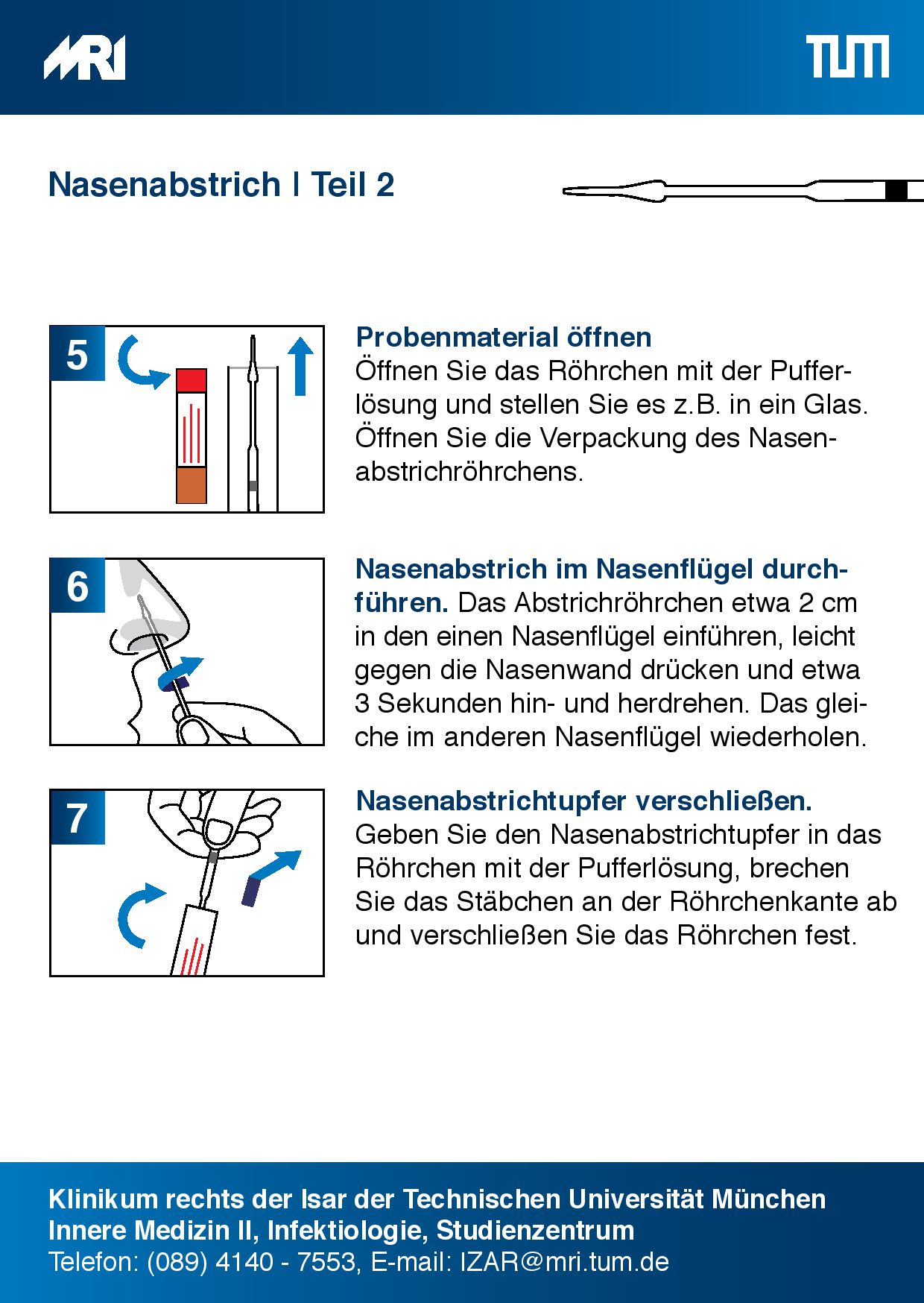

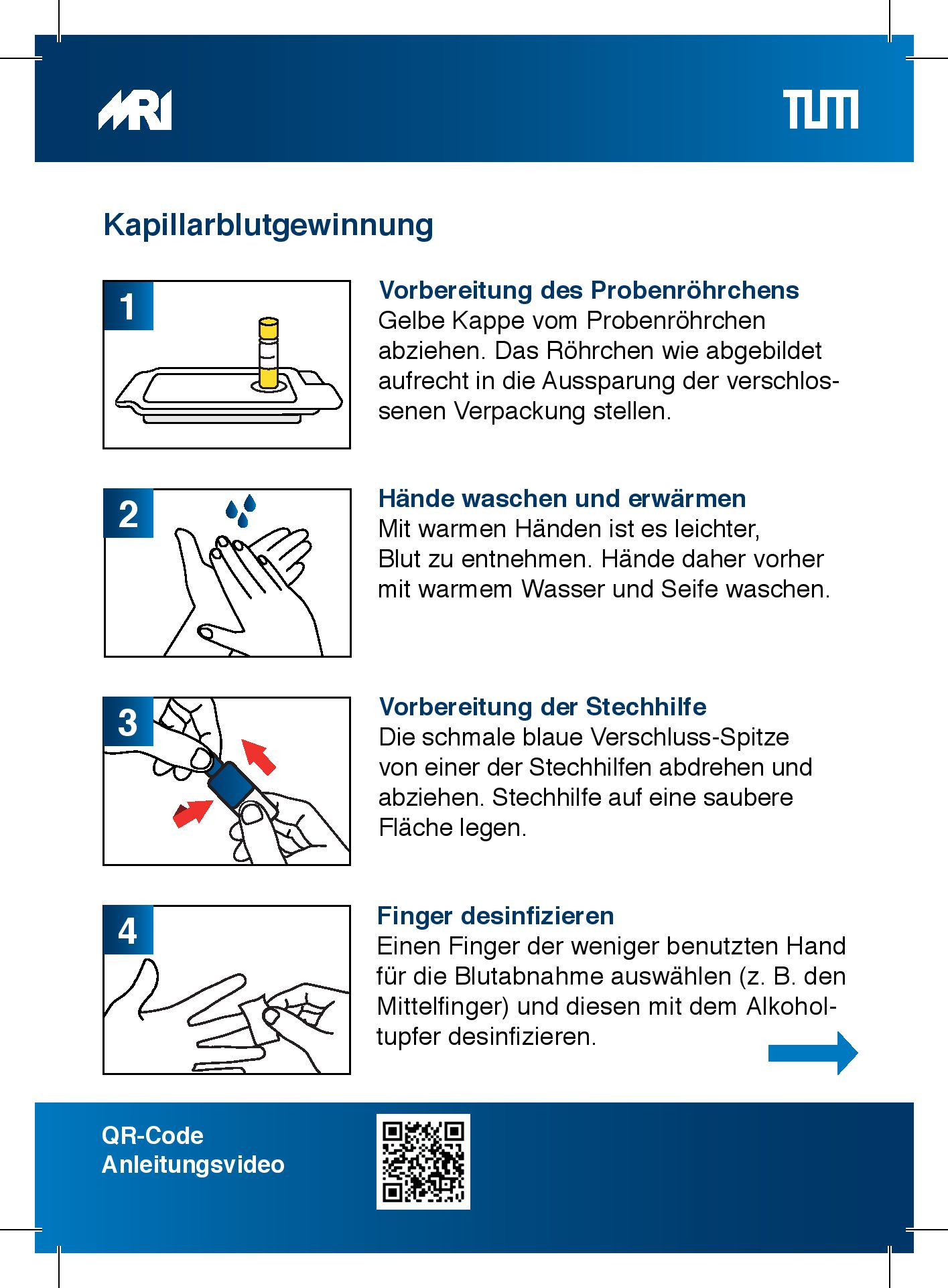

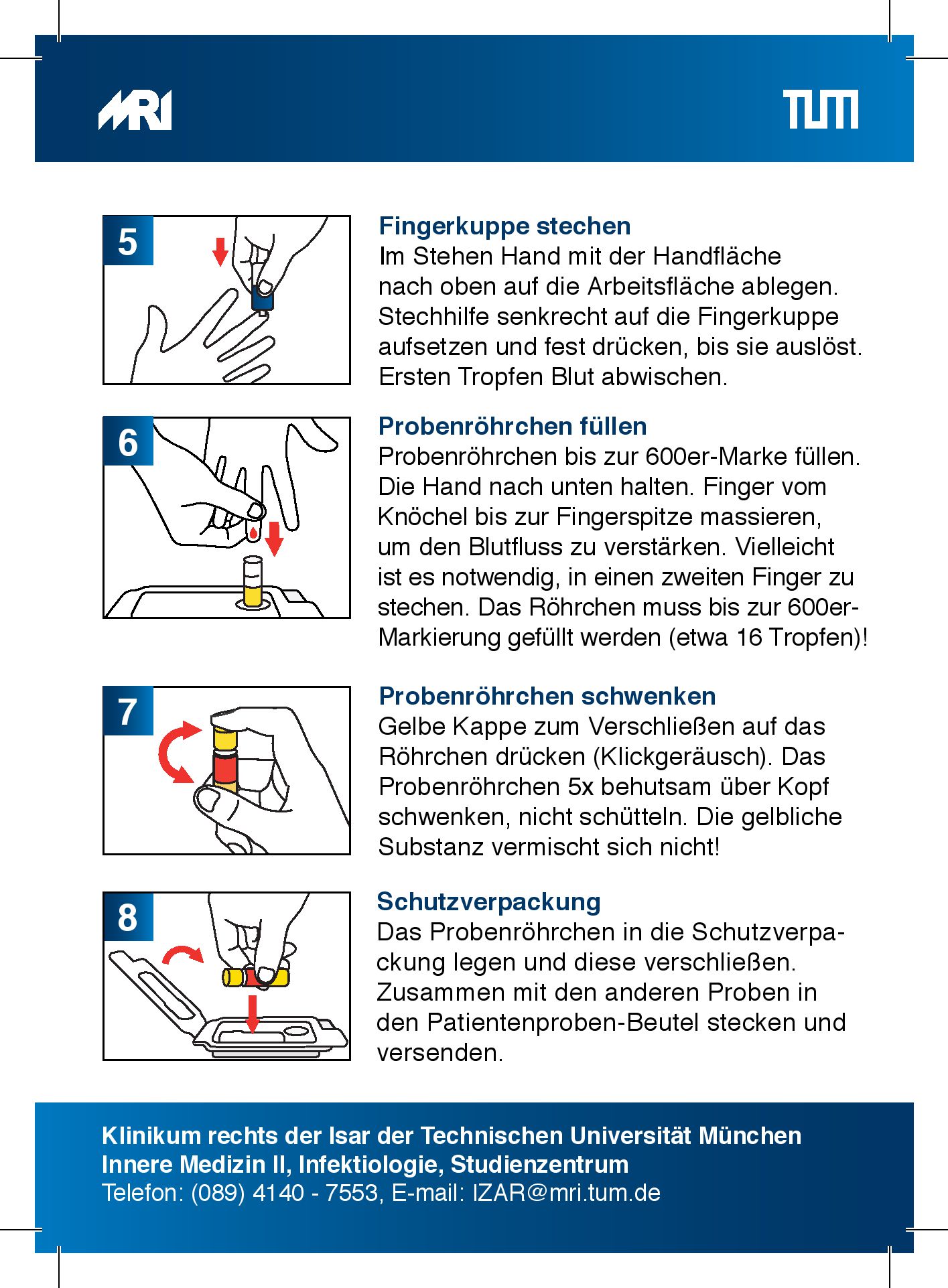


Supplementary File 1. Economical evaluation
